# Spatiotemporal ultrasound-driven piezoelectric electro-NO therapy restores neuro-vascular-immune homeostasis for radiation ulcer repair

**DOI:** 10.1016/j.mtbio.2026.103581

**Published:** 2026-08-18

**Authors:** Yueyue Nie, Xiaotong Zhao, Jingyi Li, Xinyu Huang, Biao Song, Manlin Xu, Zesu Wang, Peifeng Zhao, Beini Sheng, Yan Wang, Yali Jiang, Mingliang Tang, Hongmei Tang, Jihua Nie, Miao Xiao

**Affiliations:** a Department of Cardiovascular Surgery of the First Affiliated Hospital & Institute for Cardiovascular Science, Suzhou Medical College, Soochow University, Suzhou, 215000, China; b Department of Toxicology, School of Public Health, Medical College of Soochow University, Suzhou, 215000, China; c Department of Plastic and Aesthetic Surgery, The Second Affiliated Hospital of Soochow University, Suzhou Medical College, Soochow University, Suzhou, 215000, China; d Department of Radiotherapy and Oncology, The Second Affiliated Hospital of Soochow University, Suzhou, 215000, China

**Keywords:** Radiation ulcers, Injectable hydrogel, Piezoelectric stimulation, Nitric oxide therapy, Neuro-vascular-immune circuit

## Abstract

Radiation ulcers (RUs) represent a devastating and intractable complication arising from radiotherapy, driven by a self-perpetuating pathological cascade featuring persistent oxidative stress, unresolved chronic inflammation, progressive neurovascular destruction, and recurrent bacterial infection, which cannot be sufficiently mitigated by conventional single-target therapeutic interventions. Both electrical stimulation (ES) and nitric oxide (NO) therapy exhibit promising regenerative potential for tissue repair; nevertheless, traditional ES relies on invasive implanted electrodes with unsatisfactory spatial controllability, whereas exogenous NO delivery suffers from insufficient spatiotemporal precision and potential off-target cytotoxicity. Herein, we developed an injectable collagen hydrogel (CKPB) integrating with potassium sodium niobate nanoparticles loaded with an ultrasound-sensitive NO donor. Upon non-invasive ultrasound triggering, this integrated platform achieves wireless generation of localized electric fields as well as spatiotemporally tunable NO release. In a murine RUs model, CKPB/US treatment efficiently facilitated nerve regeneration, M2 macrophage polarization, angiogenesis, antioxidant defense and antibacterial properties. Such multi-faceted therapeutic effects contribute to the recovery of near-physiological epidermal architecture, improved functional blood perfusion, and strengthened overall tissue integrity. Collectively, this work establishes a noninvasive, dual-modality strategy to reconstruct neuro-vascular-immune homeostasis, offering a rational framework for coordinating multimodal signals to target complex pathological microenvironments.

## Graphical abstract

**Figure:**
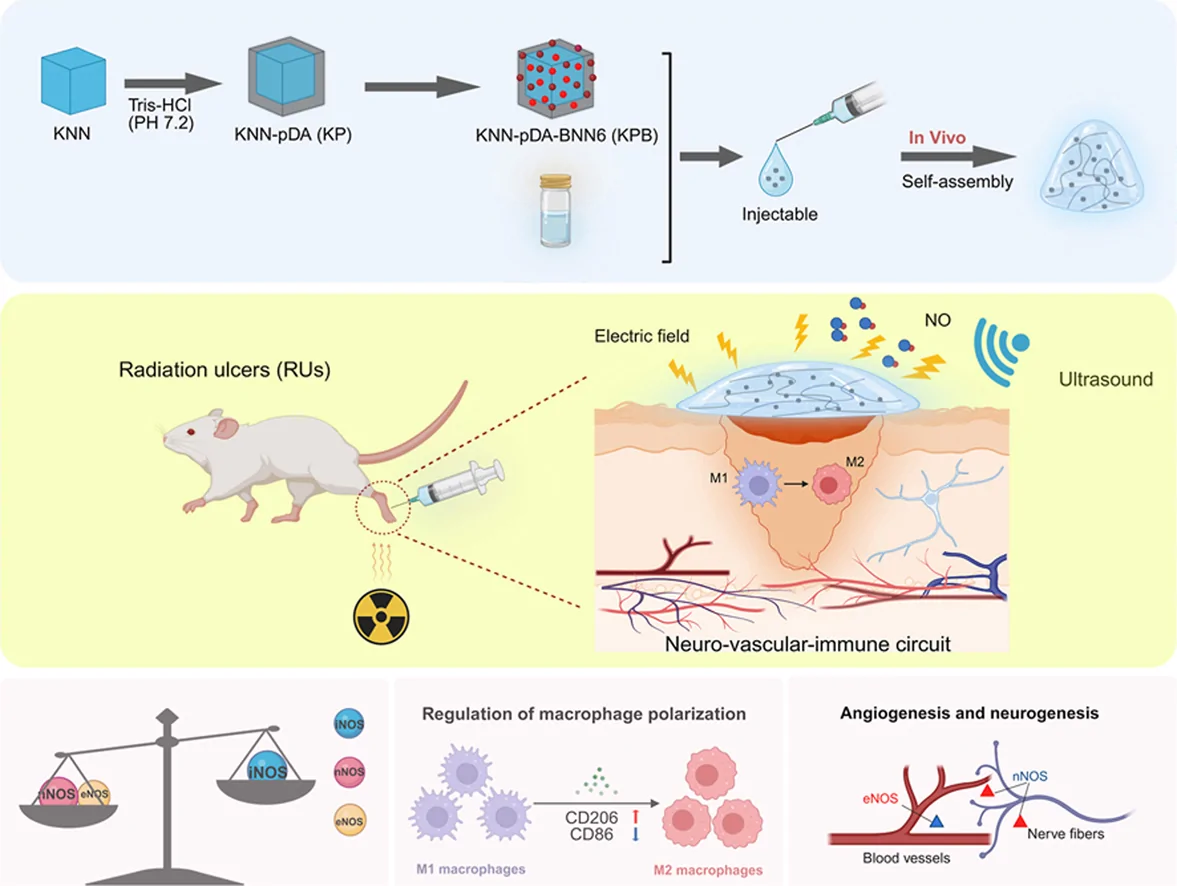

## 1 Introduction

Radiation ulcers (RUs) are debilitating complications of radiotherapy or accidental radiation exposure, affecting approximately 95% of cancer patients and leading to severe morbidity and reduced quality of life [1]. As the largest organ of the human body, the skin serves as a primary barrier against environmental insults, and its integrity is crucial for homeostasis and immune defense [2]. Unlike ordinary traumatic wounds, RUs follow a self-perpetuating destructive cascade: oxidative stress triggers DNA damage and apoptosis [3,4], M1 macrophages fuel chronic inflammation [5], vascular [3] and neural [6] disruption impairs nutrition and sensorimotor function [7,8], and a dysregulated immune microenvironment [9] sustains the cycle. Additionally, RUs are frequently complicated by bacterial infection, which aggravates tissue destruction and complicates treatment. Current clinical approaches like debridement, topical treatments, and surgical flap procedures are hindered by poor targeting, insufficient tissue integration, and failure to address the multi-dimensional pathological barriers of RUs [3]. Thus, developing a multifunctional strategy that can simultaneously modulate inflammation, promote neurovascular regeneration, scavenge oxidative stress, and inhibit infection is urgently needed.

Electrical stimulation has emerged as a promising modality for promoting tissue repair by mimicking endogenous bioelectric fields that direct cell migration, proliferation, and differentiation [10]. Electric fields stimulation can enhance fibroblast motility, facilitate endothelial tube formation, and induce macrophage polarization toward a pro-regenerative phenotype [11]. However, conventional electrotherapy typically relies on externally powered electrodes, which are often invasive, poorly localized, and associated with infection risks [12]. Smart biomaterials activated to external stimuli have revolutionized wound repair by enabling spatiotemporally controlled therapy. Among these, ultrasound (US)-activated materials are particularly attractive due to their non-invasiveness, deep tissue penetration, and clinical accessibility [13,14]. Piezoelectric materials, which convert mechanical energy into bioelectrical signals, have been shown to regulate cell behavior, promote angiogenesis, and modulate immune responses [15,16]. Among these materials, potassium sodium niobate (KNN) demonstrates strong piezoelectric properties and biocompatibility, rendering it suitable for biomedical applications [17,18].

Meanwhile, nitric oxide (NO) is a pleiotropic signaling molecule that mediates anti-inflammation, angiogenesis, and antibacterial activity [19], but its uncontrolled release causes tissue toxicity [20], highlighting the need for stimulus-activated delivery systems. BNN6, a US-sensitive NO donor, can release NO upon mechanical stimulation, making it an ideal candidate for controlled release [13]. However, single-function strategies (either electric field or NO) fail to address the complex pathological cascade of RUs, which requires effective regulation of multiple biological processes.

Injectable hydrogels have emerged as optimal carriers for localized therapy due to their ability to conform to irregular tissue defects, minimize invasive implantation [21], and provide a biomimetic extracellular matrix (ECM) microenvironment [22]. Collagen (Col), a natural component of the extracellular matrix, is extensively utilized in hydrogel fabrication due to its superior biocompatibility, biodegradability, and cell-adhesive properties [23]. Our previous studies demonstrated that collagen hydrogels can enhance cell proliferation and extracellular matrix deposition, thereby establishing a foundation for ulcer repair [24,25]. However, pure collagen hydrogels lack stimulus activation and functional bioactive components, which limit their therapeutic efficacy for challenging wounds such as RUs.

Inspired by the above challenges and opportunities, we developed a US-activated dual-functional injectable collagen hydrogel (CKPB) by integrating pDA-modified KNN NPs loaded with BNN6 into shad-derived type I collagen. The design rationale is three-fold. Firstly, the injectability and tissue adhesion of CKPB allows it to seamlessly conform to and fill irregular RUs defects. Secondly, ultrasound activation confers dual functions of​ generating a local piezoelectric electric field and enabling controllable NO release. Finally, it orchestrates effective regulation of the neurovascular-immune homeostasis. We hypothesize that this composite system can concurrently modulate neurovascular regeneration and immune homeostasis, thereby interrupting the detrimental cycle of radiation-induced ulcers. Through both *in vitro* and *in vivo* validation, we demonstrate that the CKPB significantly enhances ulcer repair, angiogenesis, nerve regeneration, and macrophage polarization, highlighting its potential as a versatile therapeutic platform for RUs.

## 2 Results

### 2.1 Characterization of the nanoparticles

**Fig. 1:**
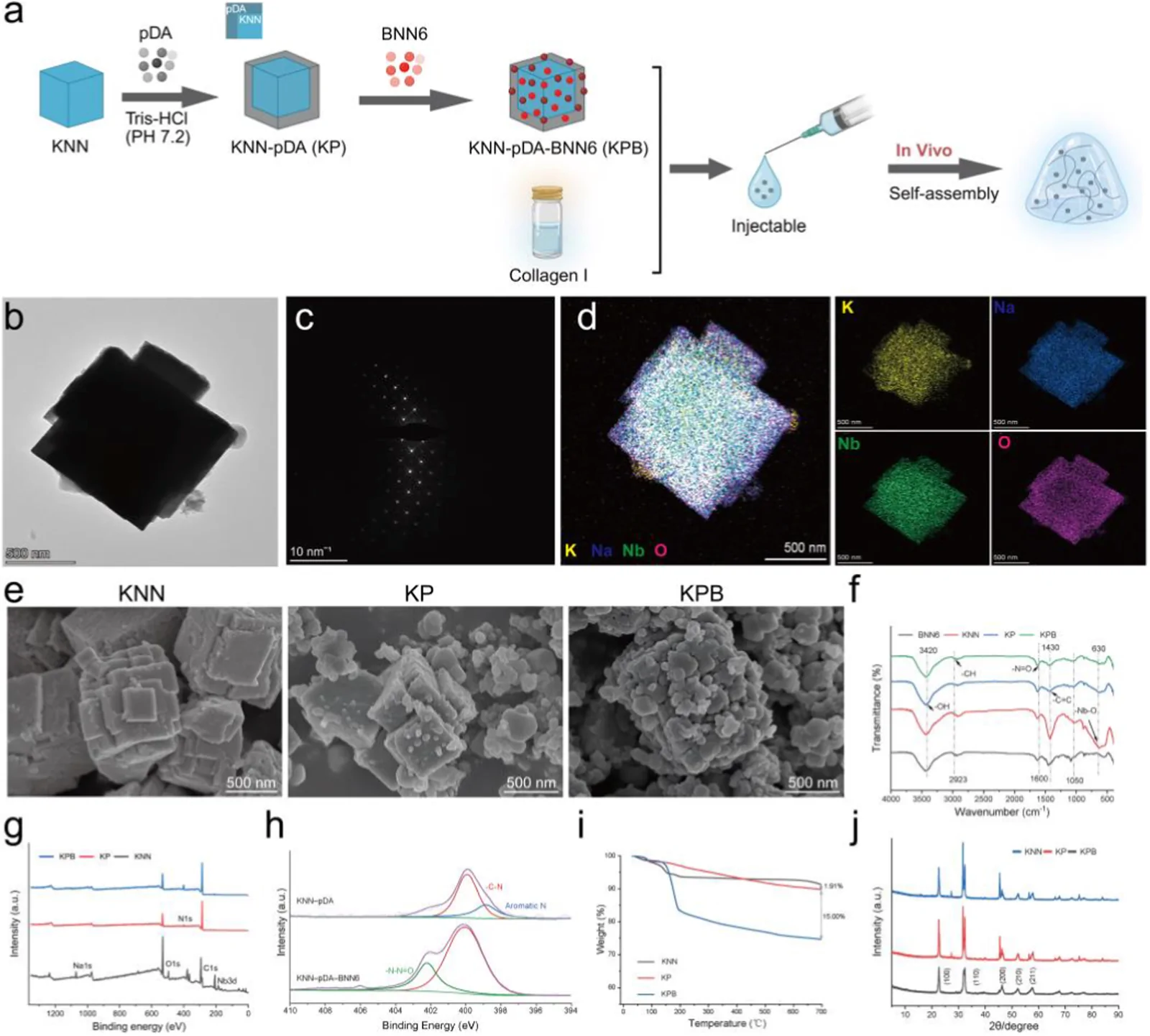
**Characterization of nanoparticles.** (a) Preparation scheme of the CKPB. (b) Representative TEM image of KNN. Scale bar = 500 nm. (c) Representative SAED image of KNN. Scale bar = 10 nm^−1^. (d) Corresponding enlarged energy-dispersive X-ray spectroscopy mapping images of KNN. Yellow, blue, green, and red represent potassium, sodium, niobium, and oxygen respectively. The experiments were repeated three times independently with comparable results. (e) Representative SEM image of KNN, KP, and KPB. Scale bar = 500 nm. (f) FTIR spectra of KNN, KP, and KPB showing shifts and new peaks indicative of hydrogen bonding and intermolecular interactions. (g) Full XPS scanning spectra of KNN, KP, and KPB. XPS revealed the subsequent disappearance of Nb 3d peak, confirming the pDA coating on the surface of KNN and the rise of N 1s peak, confirming the BNN6 coating on the surface of KP. (h) Deconvoluted high-resolution XPS N 1s spectra of the KP and KPB. (i) TGA curves of KNN, KP, and KPB. Five milligrams of each sample was loaded and heated from room temperature to 700 °C at a rate of 5 °C/min under N_2_ atmosphere. (j) The crystallographic structure of the nanoparticle determined by XRD analysis. The vacuum-dried samples were deposited on a SiO_2_ wafer with a scanning rate at 2 °C/min. The grey-colored area represents the characteristic peak splitting for an orthorhombic potassium sodium niobate at 2θ ≈ 32.8°.

To establish a functional nanoplatform for integrated function, we engineered a composite nanoparticle system through sequential surface modification of KNN. nanoparticles. Poly-dopamine (pDA) was first coated onto pristine KNN (denoted KP), followed by conjugation of the nitric oxide donor BNN6 to form the final KPB complex (KNN-pDA-BNN6) (Fig. 1a). Comprehensive characterization validates the successful fabrication and preserves crystalline integrity of the hybrid nanostructure. Transmission electron microscopy (TEM) images revealed that pristine KNN nanoparticles exhibit well-crystallized blocky morphology characteristic of orthorhombic perovs. kite (Fig. 1b). Selected-area electron diffraction (SAED) patterns further confirmed their single-crystalline nature with distinct diffraction spots (Fig. 1c). Elemental mapping demonstrated homogeneous spatial distribution of K, Na, Nb, and O, confirming structural uniformity (Fig. 1d). SEM characterizations revealed a stepwise morphological evolution of KNN upon sequential pDA and BNN6 modification: pristine KNN exhibited large blocky aggregates, KP formed rough nanoclusters, and KPB presented compact agglomerations (Fig. 1e). This progressive restructuring directly confirms effective surface functionalization. The successful fabrication of KP and KPB were further validated by Fourier-transform infrared (FTIR) and X-ray photoelectron spectroscopy (XPS) (Fig. 1f–h). The KP curve in FTIR indicated a significant decrease in native KNN peaks alongside the emergence of a characteristic benzene ring skeletal vibration at 1430 cm^−1^ (-C=C bond), unambiguously indicating pDA integration. Additionally, the KPB curve demonstrated that BNN6 was effectively loaded onto the KP surface as evidenced by the peaks at 2923 cm^−1^ corresponding to the -N=O bond [15]. A N 1s peak which was absent in pristine KNN, emerged in KP (attributed to pDA-derived nitrogen) and intensified in KPB. Concurrently, the Nb 3d peak intensity decreased in KP and was nearly undetectable in KPB, while a new N 1s peak for BNN6's N-nitroso group (-N-NO at 402 eV) appeared, accompanied by an increase in the integration value of the -C-N peak. Together, these signals confirm BNN6 immobilization on KP. Thermogravimetric analysis (TGA, Fig. 1i) quantified the mass loadings of pDA and BNN6 as 1.9 wt% and 15 wt%. Crucially, X-ray diffraction (XRD, Fig. 1j) demonstrated preserved crystal structure [17,18]. Diffraction peak positions matched the orthorhombic perovskite phase of KNN across all samples, confirming that pDA/BNN6 modification did not alter KNN's lattice integrity. A progressive decrease in diffraction peak intensity from KNN to KP to KPB was observed, attributed to X-ray shielding by the amorphous organic coatings (pDA/BNN6) on the KNN core providing orthogonal evidence of successful surface modification. Comprehensive characterization validates the successful fabrication and preserves crystalline integrity of the hybrid nanostructure.

### 2.2 Characterization of the hydrogels and nanoparticle functionality in response to ultrasound

**Fig. 2:**
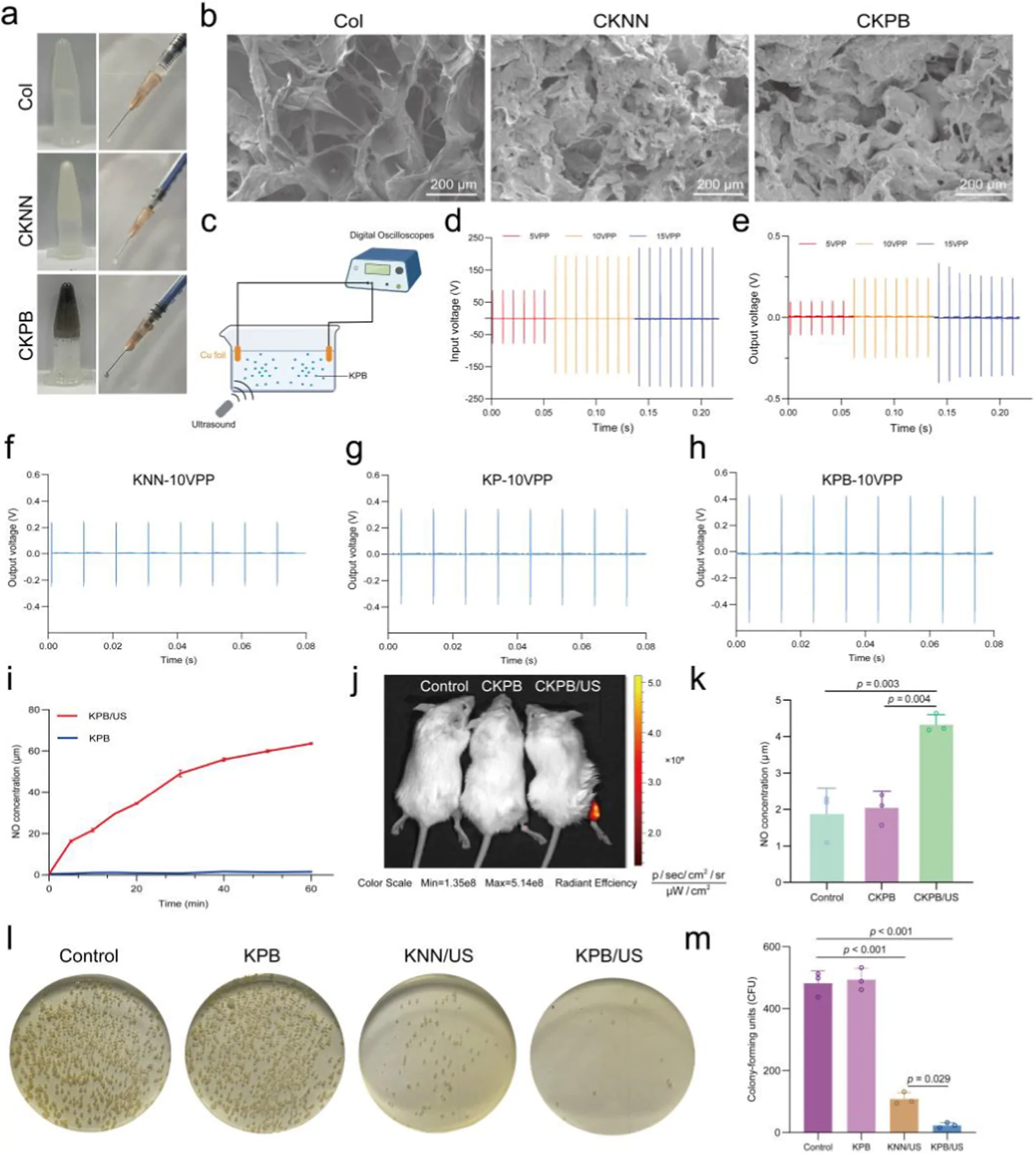
**Characterization of the injectable hydrogel and nanoparticle functionality in response to ultrasound.** (a) Col, CKNN, and CKPB solutions after PBS-induced gelation and the injectability of the hydrogels demonstrated by smooth extrusion through a syringe. (b) Representative SEM image of Col, CKNN, and CKPB. Scale bar = 200 μm. (c) Schematic diagram for testing piezoelectric performance. (d) Input and output voltages of KPB under 5 VPP, 10 VPP, and 15 VPP. (e) Output voltages of KNN under 10 VPP. (f) Output voltages of KP under 10 VPP. (g) Output voltages of KPB under 10 VPP. (i) The real-time cumulative NO release profile of the nanoparticles, in response to the continuous ultrasound exposure *in vitro*. (j) Representative image of NO release detected by NO fluorescent probes *in vivo*. (k) Quantification of NO in skin tissues. Data are presented as mean ± SD (n = 3). (l) Representative images of bacterial colonies cultured in the Control group, KPB group, KNN combined with ultrasound (KNN/US) group, and KPB combined with ultrasound (KPB/US) group; the reduction in colony number reflects the antibacterial efficacy of the samples. (m) Quantitative analysis of antibacterial activity via colony-forming unit (CFU) counting statistical results of bacterial CFU in the Control, KPB, KNN/US, and KPB/US groups. Data are presented as mean ± SD (n = 3).

To establish an injectable theragnostic platform with spatiotemporal controllability, we engineered collagen-based hydrogels integrated with functionalized nanoparticles capable of dual-response to ultrasound excitation. To validate the core functionalities of CKPB, we conducted the following experiments. Fig. 2A demonstrates the *in-situ gelation* of Col, CKNN, and CKPB hydrogels triggered by physiological ionic/temperature cues (PBS, 37 °C). Crucially, all precursor solutions maintained injectable liquid states pre-injection, enabling smooth extrusion through needles without clogging. Upon subcutaneous administration in mice, these formulations underwent rapid sol-gel transition under physiological conditions, forming stable hydrogels readily distinguishable from surrounding tissues (Supplementary Fig. S1). This confirms their dual-triggered (ionic/thermal) *in vivo* self-assembly capability for minimally invasive targeted therapy. SEM analysis revealed that pure collagen scaffolds possessed a highly porous interconnected architecture. While minor structural changes occurred upon incorporating KNN and KPB nanoparticles, the composite hydrogels maintained essential porosity (Fig. 2b).

To validate piezoelectric functionality, KPB nanoparticles were immersed in PBS positioned 2 cm from an ultrasonic transducer with a diameter of 4 cm, with generated electrical signals monitored under sonication (Fig. 2c and Supplementary Fig. S2). The output voltage of KPB increases with the rise of input voltage and peak-to-peak voltage which were from 0.1 to 0.35 V (Fig. 2d, e). Under the same input voltage and peak-to-peak voltage conditions, the output voltages of KNN, KP, and KPB maintained stable piezoelectric output (0.2-0.4 V; Fig. 2f–h). NO release kinetics were comprehensively evaluated through integrated *in vitro* and *in vivo* analyses, where Griess assays demonstrated ultrasound-triggered enhancement of *in vitro* NO release with progressive temporal accumulation versus non-sonicated controls (Fig. 2i). *In vivo*, fluorescence imaging revealed distinct spatial distribution patterns: Control and CKPB groups exhibited minimal orange-yellow fluorescence (basal NO levels), while CKPB/US showed intense signals with expanded distribution areas (Fig. 2j). Quantitative Griess assays of dermal tissues confirmed significantly elevated NO in CKPB/US (4.360 ± 0.274 μM) compared to control (1.913 ± 0.711 μM; *p* = 0.003) and CKNN (2.078 ± 0.462 μM; *p* = 0.004), with CKPB displaying marginally higher NO (2.410 ± 0.381 μM) than control without statistical significance, collectively establishing that ultrasound-activated CKPB achieves 2.3-fold higher NO generation than non-sonicated systems, demonstrating spatiotemporally controllable therapeutic delivery. Moreover, according to the result of antibacterial assay (Fig. 2l, m), KPB/US demonstrated potent bactericidal efficacy, achieving a 95% kill/elimination rate (vs. Control, *p* < 0.001). These results confirm that the injectable CKPB exhibits ultrasound-responsive piezoelectricity and NO release as well as potent antibacterial activity, laying a solid foundation for subsequent verification of its role in promoting RUs healing.

### 2.3 Therapeutic efficacy of CKPB/US on RUs *in vivo*

**Fig. 3:**
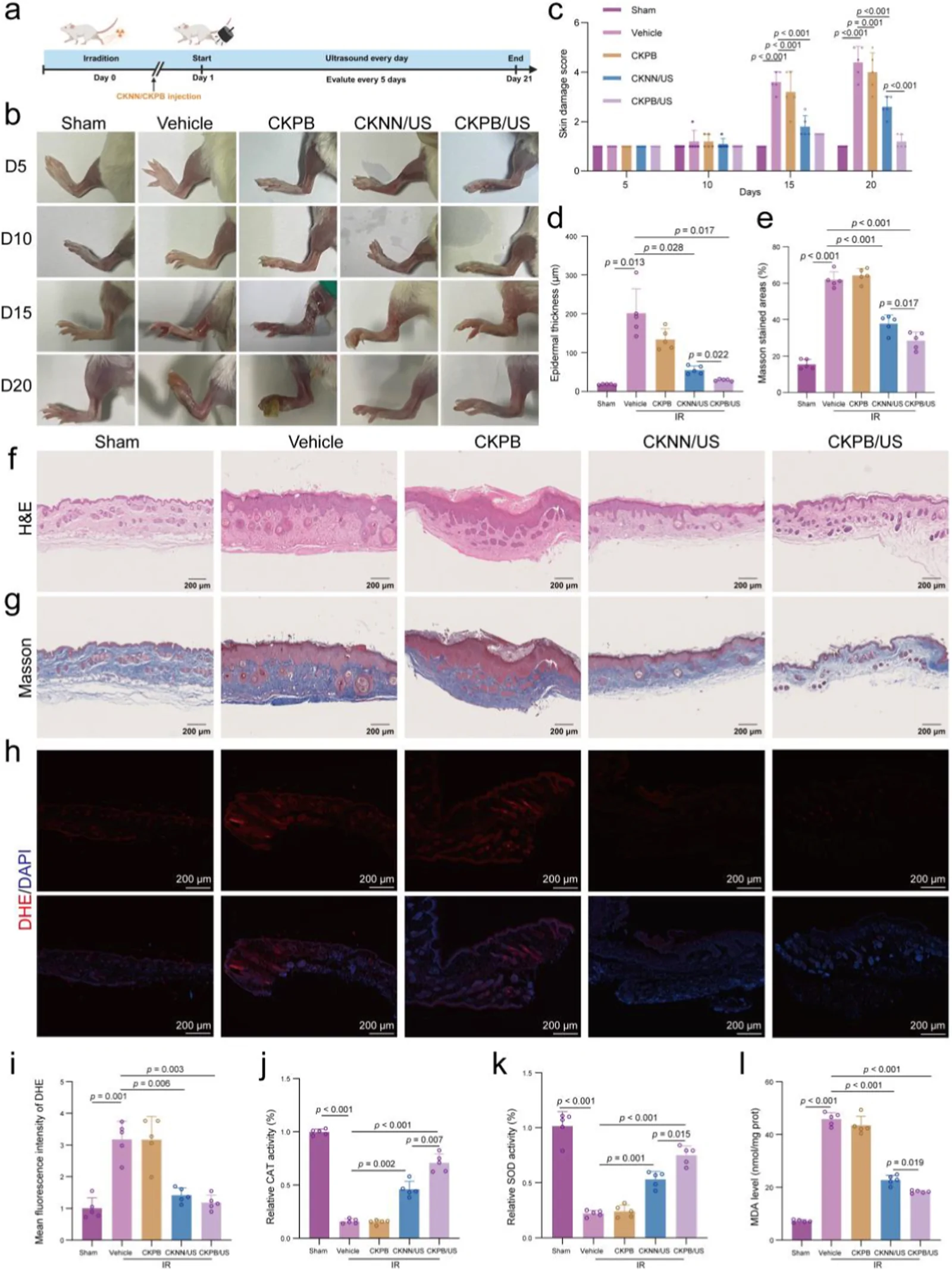
**Therapeutic efficacy of US triggered localized piezoelectric electric fields and spatiotemporally controlled NO release on RUs *in vivo*.** (a) Schematic diagram of the *in vivo* animal experimental protocol. (b) Representative images on days 5, 10, 15, and 20 post-irradiation showing accelerated ulcer healing in the CKPB/US group compared with Sham, Vehicle, CKPB, and CKNN/US groups. (c) Clinical pathology scores over the 20-day period showing reduced tissue damage in the CKPB/US group (n = 5). Data are presented as mean ± SD. (d) Epidermal thickness measurements indicating increased epidermal regeneration in the CKPB/US group (n = 5). Data are presented as mean ± SD. (e) Quantification of collagen deposition from Masson-stained sections showing enhanced matrix remodeling in the CKPB/US (n = 5). The data are showed with mean ± SD. (f) Representative images of H&E indicating improved epidermal regeneration in the CKPB/US group. Scale bar = 200 μm. (g) Representative images of Masson's trichrome staining indicating improved organized collagen deposition in the CKPB/US group. Scale bar = 200 μm. (h) Representative images of Dihydroethidium fluorescence staining indicating reduced reactive oxygen species (ROS) levels in the CKPB/US group. Scale bar = 200 μm. (i) Quantitative analysis of DHE fluorescence intensity (n = 5). (j) Catalase (CAT) activity levels in skin tissues (n = 5). (k) Superoxide dismutase (SOD) activity levels (n = 5). (l) Malondialdehyde (MDA) content indicating lipid peroxidation levels (n = 5). The data are showed with mean ± SD.

To rule out intrinsic biological effects induced by ultrasound, we tracked hindlimb injury status in Vehicle (RUs) and Vehicle/US groups at Day 5, 10, 15 and 20 and performed quantitative skin-damage scoring (Supplementary Fig. S3a and b). Together with color Doppler based blood flow images and quantification (Supplementary Fig. S3c and d), these data confirmed that ultrasound treatment alone does not alter the pathological progression of RUs. The therapeutic potential of US triggered localized piezoelectric electric fields and spatiotemporally controlled NO release was assessed in a 21-day murine RUs model (Fig. 3a). To verify long-term *in vivo* NO release, CKPB hydrogel was injected into mouse hindlimbs for sequential photographic documentation (Fig. S4a and b). NO release upon ultrasound stimulation was evaluated at days 7, 14, and 21 post-implantation via small-animal in-vivo imaging and NO assay kits. Quantitative NO measurements revealed persistently elevated NO concentrations in the CKPB group relative to controls across all three time points (Fig. S4d). Consistently, ultrasound-triggered NO-associated signals were detectable at all monitored time points by in-vivo imaging (Fig. S4c). Sustained NO production even at day 21 confirms that implanted CKPB retains responsive capability under ultrasound stimulation in living tissue.

During days 5-20 post-irradiation, the hind limb skin of the Vehicle group displayed progressive RUs manifestations including persistent hair loss, escalating erythema, desquamation, and ultimately refractory skin ulcers with dermal necrosis (Fig. 3b). To quantify the damage status of RUs, we performed a grading assessment based on previous studies [26]. In contrast, all treatment groups exhibited less severe ulceration than the Vehicle group, notably with CKPB/US presenting the mildest pathology. These findings demonstrate that CKPB/US significantly enhanced ulcer repair. Clinical pathology scores on day 20 confirmed superior healing efficacy in the CKPB/US group (1.430 ± 0.451) versus Vehicle controls (4.440 ± 0.560; *p* < 0.001), establishing its therapeutic advantage (Fig. 3c). Skin epidermal thickening and excessive collagen deposition drive scar hyperplasia which is a prevalent complication in RUs that impairs wound healing [27]. Notably, the CKPB/US group exhibited significant improvement in both pathological features (Fig. 3f, g). Quantitative analyses confirmed these findings. H&E staining revealed epidermal thickness in the CKPB/US group (29.520 ± 2.541 μm) was comparable to the Sham group (18.200 ± 1.300 μm) and significantly reduced versus the Vehicle group (201.600 ± 62.950 μm; *p* = 0.017; Fig. 3d). Masson staining demonstrated markedly lower collagen deposition in the CKPB/US group (28.550 ± 4.700%) relative to Vehicle controls (61.920 ± 4.411%; *p* < 0.001; Fig. 3e). Critically, these morphological improvements resulted in restoration of the skin's native architecture.

Moreover, DHE fluorescence staining was utilized to assess CKPB/US effects on oxidative stress in skin tissue. The analysis revealed significantly attenuated ROS levels in the CKPB/US group (1.181 ± 0.239 AU) versus the Vehicle group (3.181 ± 0.568 AU; *p* = 0.003), with values approaching physiological baselines observed in the Sham group (1.000 ± 0.330 AU) (Fig. 3h, i). To delineate the underlying antioxidant mechanisms, we quantified key enzymatic activities [28]. SOD activity was markedly enhanced by CKPB/US (0.751 ± 0.085) relative to Vehicle (0.222 ± 0.028; *p* < 0.001; Fig. 3j). CAT activity showed parallel improvement (0.709 ± 0.084 vs. Vehicle 0.161 ± 0.022; *p* < 0.001; Fig. 3k). Notably, both enzymes were restored to near-Sham levels.

Lipid peroxidation (measured via MDA) was substantially reduced in the CKPB/US group (18.26 ± 0.504 nmol/mg prot) compared to Vehicle (45.84 ± 2.459 nmol/mg prot; *p* < 0.001; Fig. 3l). Collectively, the CKPB/US group exhibited markedly enhanced antioxidant capacity.

### 2.4 CKPB/US enhanced vascular regeneration *in vivo*

**Fig. 4:**
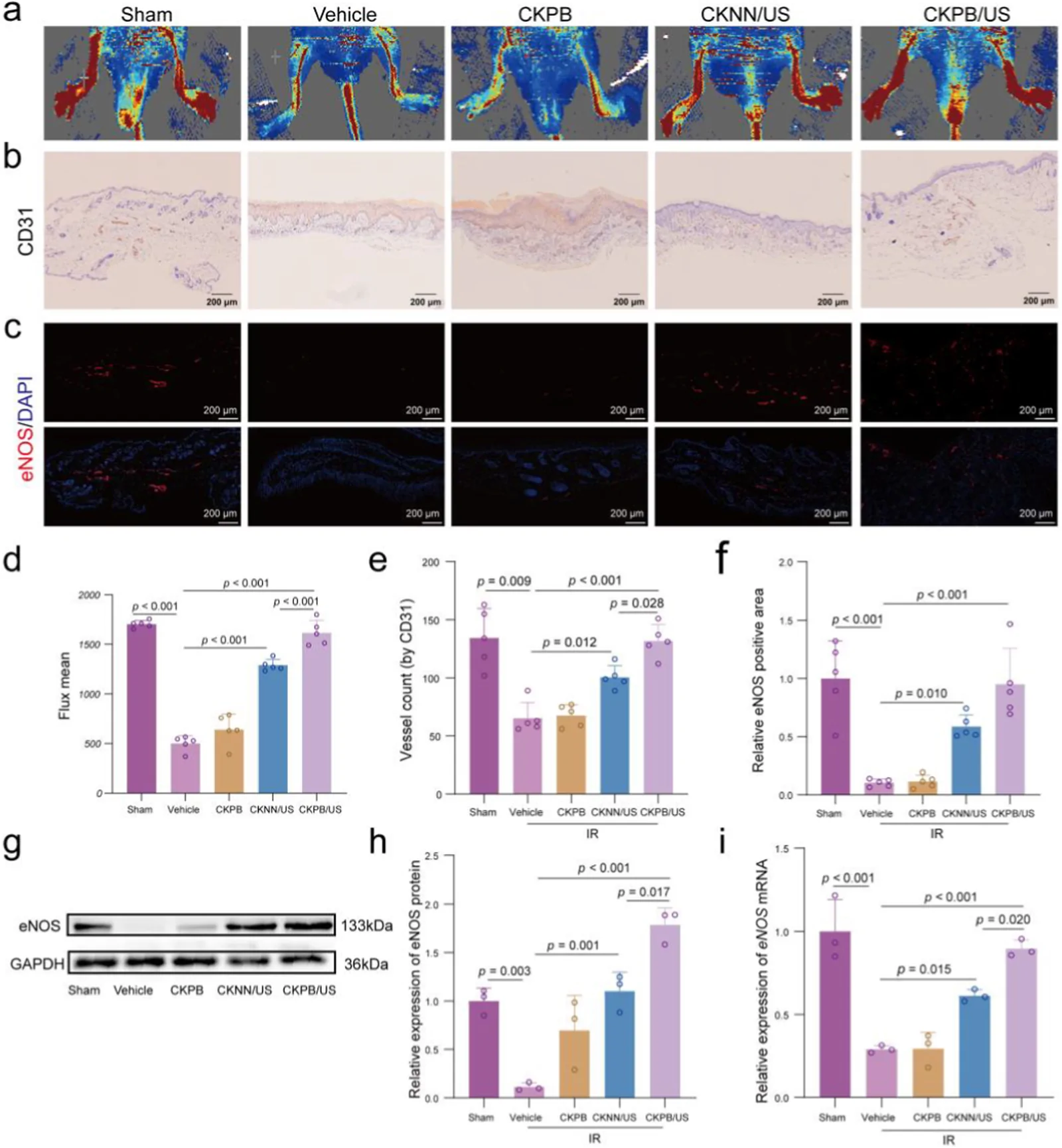
**CKPB/US enhanced vascular regeneration *in vivo*.** (a) Color Doppler imaging on day 21 indicating enhanced blood perfusion in the CKPB/US group. (b) Immunohistochemical staining of CD31 indicating enhanced vascularization in the CKPB/US group. Scale bar = 200 μm. (c) Immunofluorescence staining for endothelial nitric oxide synthase (eNOS, red) and nuclei (DAPI, blue); Merge indicates the merged images. Scale bar = 200 μm. (d) Quantitative Doppler analysis of blood flow indicating elevated perfusion in the CKPB/US group (n = 5). The data are showed with mean ± SD. (e) Quantification of CD31^+^ vessel (n = 5). Data are presented as mean ± SD. (f) Quantification of eNOS-positive per unit area (n = 5). (g) Western blot analysis of eNOS protein levels. (h) Densitometric quantification of Western blot bands for eNOS (n = 3). (i) Relative mRNA expression of eNOS (n = 3).

The healing of RUs is critically dependent on the successful regeneration of functional blood vessels, a process known as angiogenesis [29]. Chronic radiation damage devastates the local microvasculature, leading to tissue ischemia, hypoxia, and a persistent state of inflammation that characterizes these stubborn ulcers. By restoring adequate blood flow, such approaches can reverse the ischemic drive of the ulcer, facilitate the delivery of reparative cells, and finally shift the pathological microenvironment toward one conducive to granulation tissue formation and re-epithelialization [30]. To explore the impact of CKPB/US on vascular and epidermal nerve regeneration in RUs, laser Doppler blood flowmetry was used to assess lower limb blood perfusion. Robust blood flow signals were observed in the Sham group (1704 ± 36.670), whereas the Vehicle group showed marked perfusion reduction, with the CKPB/US group exhibiting significant perfusion recovery (Fig. 4a). Quantitative analysis of mean lower limb blood flow corroborated this recovery (Vehicle 500.600 ± 77.882 vs. CKPB/US 1616.000 ± 126.400; *p* < 0.001; Fig. 4d), demonstrating that CKPB/US mitigates radiation-induced skin blood perfusion deficits. The expression level of CD31 shows a positive correlation with the degree of angiogenesis [31]. For angiogenesis assessment, the immunohistochemistry of CD31 revealed sparse CD31^+^ vascular structures in the Vehicle group, while the CKPB/US group displayed increased the number of CD31^+^ vessel (Vehicle 60.60 ± 16.33 vs. CKPB/US 134.8 ± 14.13; *p* < 0.001; Fig. 4b–e). Immunofluorescence staining for endothelial nitric oxide synthase (eNOS), a key mediator of angiogenesis [32], showed weak fluorescence intensity in the Vehicle group, whereas the CKPB/US group had significantly enhanced eNOS expression (Vehicle 0.104 ± 0.032 vs. CKPB/US 0.951 ± 0.306; *p* < 0.001; Fig. 4c–f). Western blot analysis quantified protein levels of eNOS (Fig. 4g). The Vehicle group showed downregulated eNOS (Sham 1.790 ± 0.176 vs. Vehicle 0.114 ± 0.040; *p* < 0.001; Fig. 4h), while the CKPB/US group significantly upregulated these proteins with a more pronounced effect than the CKPB and CKNN/US groups. Quantitative real-time PCR further confirmed that CKPB/US upregulated eNOS mRNA levels in RUs (0.897 ± 0.052 vs. Vehicle 0.290 ± 0.023; *p* < 0.001; vs. CKNN/US 0.613 ± 0.037; *p* = 0.002; Fig. 4i), indicating that CKPB/US promotes angiogenesis. Collectively, these findings demonstrate that CKPB/US treatment significantly promotes angiogenesis in radioactive skin ulcers by restoring blood perfusion, increasing microvascular density, and upregulating key pro-angiogenic factors like eNOS. This robust vascular regeneration underscores the therapeutic potential of CKPB/US for improving healing in radiation-damaged skin.

### 2.5 CKPB/US promoted epidermal nerve regeneration *in vivo*

**Fig. 5:**
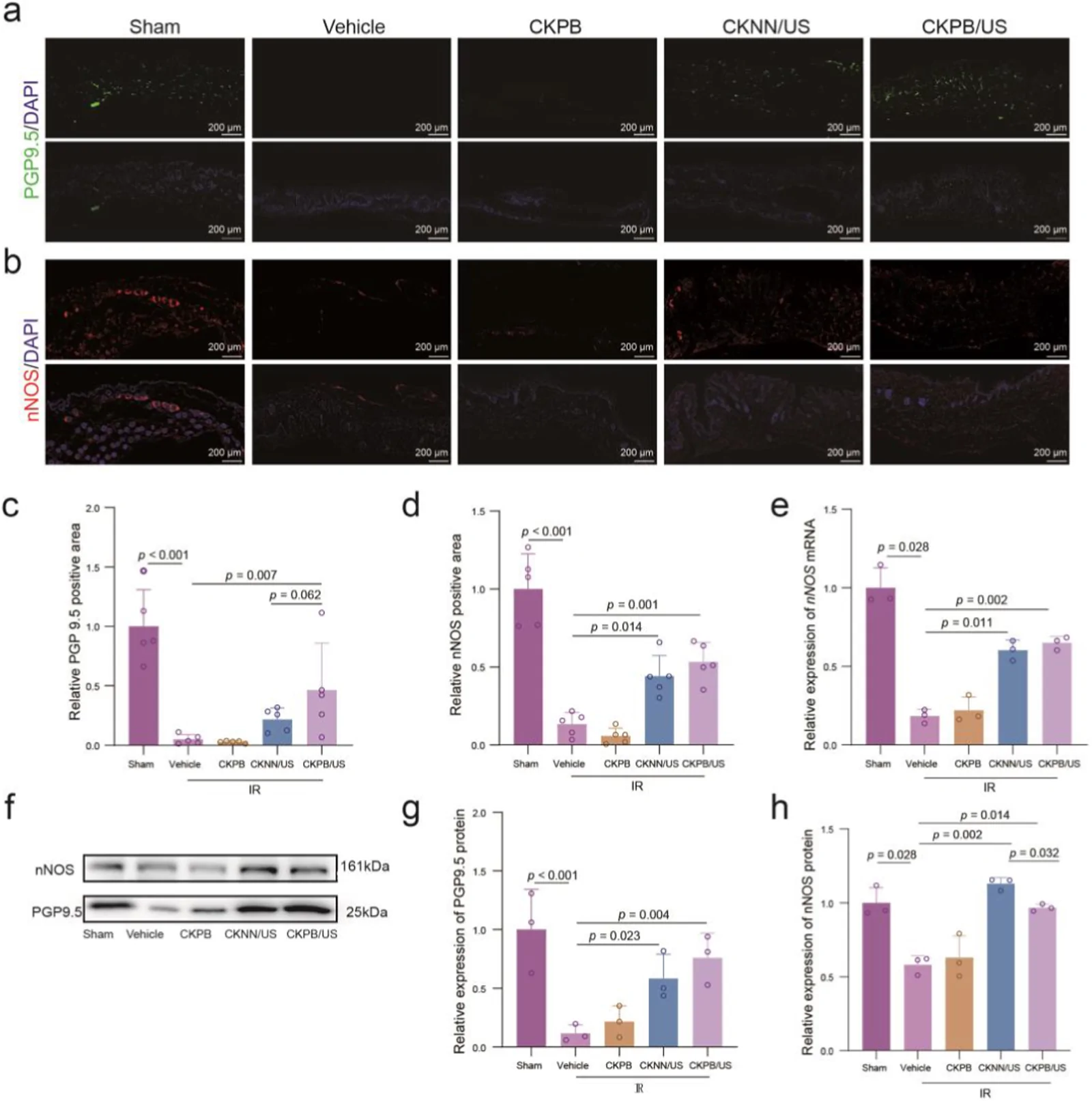
**CKPB/US promoted epidermal nerve regeneration *in vivo*.** (a) Immunofluorescence staining for the neural marker PGP9.5 (green) and nuclei (DAPI, blue); Merge indicates the merged images. Scale bar = 200 μm. (b) Immunofluorescence staining for neuronal nitric oxide synthase (nNOS, red) and nuclei (DAPI, blue); Merge indicates the merged images. Scale bar = 200 μm. (c) Quantification of PGP9.5-positive per unit area (n = 5). (d) Quantification of nNOS-positive per unit area (n = 5). (e) Relative mRNA expression of nNOS (n = 3). (f) Western blot analysis of PGP9.5 and nNOS protein levels. GAPDH loading control was shown in Fig. 4g. (g) Densitometric quantification of Western blot bands for PGP9.5 (n = 3). (h) Densitometric quantification of Western blot bands for nNOS (n = 3).

Beyond the vascular component, the regeneration of peripheral nerves plays an equally vital and often overlooked role in the healing of radioactive skin ulcers. The skin is a highly innervated organ, and neurogenic factors released by nerve endings are crucial for modulating local inflammation, promoting cell proliferation, and maintaining tissue homeostasis. Radiation-induced nerve damage disrupts this delicate neurocutaneous signaling, impairing the ulcer's innate healing capacity. Regarding epidermal nerve regeneration, immunofluorescence for protein gene product 9.5 (PGP9.5), a pan-neuronal marker [13,33], revealed drastically reduced signals in the Vehicle group, with the CKPB/US group showing restored PGP9.5 integrated intensity (0.465 ± 0.394 vs. Vehicle 0.051 ± 0.039; *p* = 0.007; Fig. 5a–c). For neuronal nitric oxide synthase (nNOS), a marker of peripheral nerve function, the Vehicle group had diminished nNOS fluorescence, while the CKPB/US group exhibited elevated nNOS intensity (0.532 ± 0.125 vs. Vehicle 0.132 ± 0.075; *p* = 0.001; Fig. 5b–d); qPCR verified that CKPB/US upregulated nNOS mRNA expression (0.649 ± 0.042 vs. Vehicle 0.183 ± 0.044; *p* = 0.002; Fig. 5e), confirming its ability to facilitate epidermal nerve regeneration in RUs. Western blot analysis quantified protein levels of nNOS, and PGP9.5 (Fig. 5f). The Vehicle group showed downregulated nNOS (0.968 ± 0.024 vs. Vehicle 0.580 ± 0.062; *p* = 0.014; Fig. 5g), and PGP9.5 (1.320 ± 0.023 vs. Vehicle 0.490 ± 0.163; *p* = 0.004; Fig. 5h), while the CKPB/US group significantly upregulated these proteins with a more pronounced effect than the CKPB and CKNN/US groups. These findings confirm that CKPB/US effectively facilitates the restoration of peripheral nerve innervation, which is crucial for achieving functional healing of RUs.

### 2.6 CKPB/US alleviated RUs by regulating macrophage polarization from M1 to M2

**Fig. 6:**
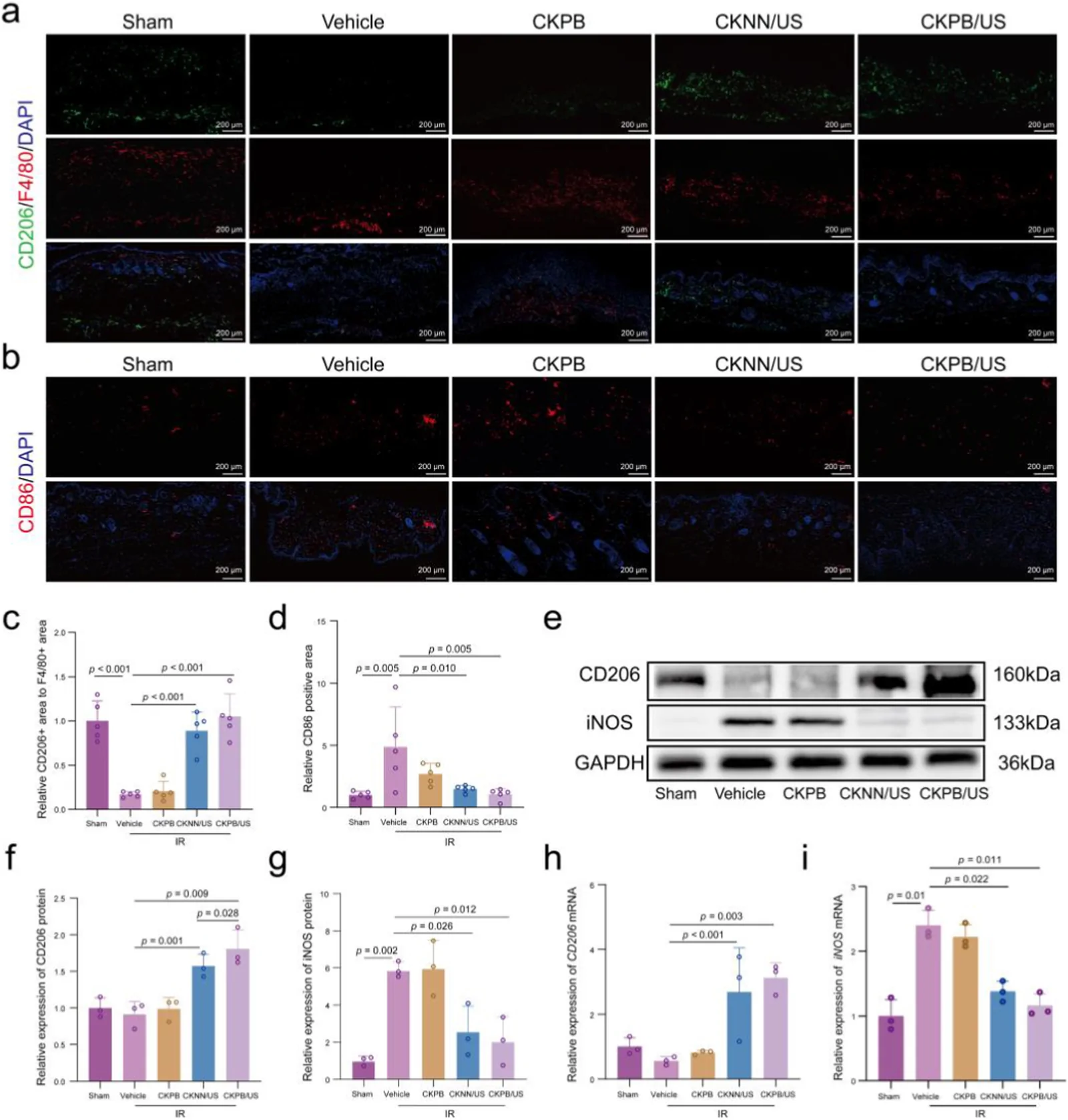
**CKPB/US alleviated RUs by regulating macrophage polarization from M1 to M2.** (a) Immunofluorescence staining for F4/80 (red), CD206 (green) and nuclei (DAPI, blue); Merge indicates the merged images. Scale bar = 200 μm. (b) Immunofluorescence staining for CD86 (red) and nuclei (DAPI, blue); Merge indicates the merged images. Scale bar = 200 μm. (c) Quantification of the ratio of CD206^+^ (M2) area to F4/80^+^ (total macrophage) area (n = 5). (d) Quantification of CD86-positive per unit area (n = 5). (e) Western blot analysis of iNOS and CD206 protein levels. (f) Densitometric quantification of Western blot bands for CD206 (n = 3). (g) Densitometric quantification of Western blot bands for iNOS (n = 3). (h) Relative mRNA expression of CD206 (n = 3). (i) Relative mRNA expression of iNOS (n = 3).

Macrophage polarization, a key regulator of the inflammatory microenvironment, plays a pivotal role in orchestrating the cascade of tissue repair and regeneration processes [33]. To evaluate the regulatory effect of CKPB/US on macrophage phenotypes, we detected pan-macrophage marker F4/80, M2-specific marker CD206, and M1-specific markers CD86 via immunofluorescence staining, Western blot, and quantitative real-time reverse transcription polymerase chain reaction (for mRNA expression) [34]. Immunofluorescence staining of F4/80 (total macrophages) and CD206 (M2 macrophages) revealed the distribution of total and M2 macrophages in ulcer tissues, with the CD206^+^/F4/80^+^ ratio used to quantify M2 proportion. The CD206^+^/F4/80^+^ ratio was significantly lower in the Vehicle group than in the Sham group (Sham 1.000 ± 0.223 vs. Vehicle 0.171 ± 0.0314; *p* < 0.001), while both the CKNN/US group (Vehicle 0.171 ± 0.0314 vs. CKNN/US 0.890 ± 0.207; *p* < 0.001) and CKPB/US group (Vehicle 0.171 ± 0.0314 vs. CKPB/US 1.05 ± 0.257, *p* < 0.001) exhibited markedly increased ratios relative to the Vehicle group; no significant difference was found between the CKNN/US and CKPB/US groups (CKNN/US 0.890 ± 0.207 vs. CKPB/US 1.05 ± 0.257, *p* > 0.05; Fig. 6a–c). For CD86 staining, the relative CD86^+^ area was significantly higher in the Vehicle group than in the Sham group (Sham 1.000 ± 0.290 vs. Vehicle 4.880 ± 3.190, *p* = 0.005). Both CKNN/US (Vehicle 4.880 ± 3.190 vs. CKNN/US 1.493 ± 0.279; *p* = 0.01) and CKPB/US (Vehicle 4.880 ± 3.190 vs. CKPB/US 1.060 ± 0.449; *p* = 0.005) treatments dramatically reduced the relative CD86-positive area compared with the Vehicle group, and no statistical difference was observed between the CKNN/US and CKPB/US groups (CKNN/US 1.490 ± 0.279 vs. CKPB/US 1.060 ± 0.449; *p* > 0.05; Fig. 6b–d). Western blot analysis was performed to detect the relative expression of CD206 and iNOS proteins. For CD206 protein, the Vehicle group showed a slightly lower expression than the Sham group (Sham 1.00 ± 0.138 vs. Vehicle 0.914 ± 0.175; *p* > 0.05), while the CKNN/US group exhibited a significantly higher expression compared with the Vehicle group (Vehicle 0.914 ± 0.175 vs. CKNN/US 1.57 ± 0.163; *p* = 0.001), and the CKPB/US group also had a markedly elevated expression relative to the Vehicle group (Vehicle 0.914 ± 0.175 vs. CKPB/US 1.81 ± 0.260, *p* = 0.028); importantly, CD206 protein expression in the CKPB/US group was significantly higher than that in the CKNN/US group (CKNN/US 1.570 ± 0.163 vs. CKPB/US 1.810 ± 0.260; *p* = 0.009; Fig. 6e, f). For iNOS protein, the expression was significantly higher in the Vehicle group than in the Sham group (Sham 1.000 ± 0.246 vs. Vehicle 5.900 ± 0.421; *p* = 0.002); both CKNN/US (Vehicle 5.900 ± 0.421 vs. CKNN/US 2.530 ± 1.420, *p* = 0.026) and CKPB/US (Vehicle 5.900 ± 0.421vs. CKPB/US 2.070 ± 1.300, *p* = 0.012) significantly downregulated iNOS relative to the Vehicle group, with no statistical difference in iNOS protein levels between the two experimental groups (CKNN/US 2.530 ± 1.420 vs. CKPB/US 2.070 ± 1.300, *p* > 0.05; Fig. 6e–g). qRT-PCR was performed to assess the mRNA expression of CD206 and iNOS. For CD206 mRNA, the CKNN/US group showed a significantly higher expression compared with the Vehicle group (Vehicle 0.559 ± 0.127 vs. CKNN/US 2.690 ± 1.360; *p* < 0.001), and the CKPB/US group had a further elevated expression relative to the CKNN/US group (CKNN/US 2.690 ± 1.360 vs. CKPB/US 3.120 ± 0.466; *p* = 0.003; Fig. 6h). For iNOS mRNA, the expression was significantly higher in the Vehicle group than in the Sham group (Sham 1.000 ± 0.255 vs. Vehicle 2.400 ± 0.234; *p* = 0.01); both CKNN/US (Vehicle 2.40 ± 0.234 vs. CKNN/US 1.38 ± 0.160; *p* = 0.022) and CKPB/US (Vehicle 2.400 ± 0.234 vs. CKPB/US 1.160 ± 0.180; *p* = 0.011) significantly downregulated iNOS mRNA relative to the Vehicle group, and the CKPB/US group exhibited a slight but non-significant lower expression than the CKNN/US group (CKNN/US 1.380 ± 0.160 vs. CKPB/US 1.160 ± 0.180; *p* > 0.05; Fig. 6i).

Collectively, these observations clearly establish that both CKNN/US and CKPB/US can promote the transition of macrophages from the M1 to M2 phenotype, with CKPB/US exhibiting superior regulatory activity, evidenced by significantly higher CD206 protein (CKNN/US 1.570 ± 0.163 vs. CKPB/US 1.810 ± 0.260; *p* = 0.009) and mRNA (CKNN/US 2.690 ± 1.360 vs. CKPB/US 3.120 ± 0.466; *p* = 0.003) expression, as well as more pronounced downregulation of iNOS mRNA compared with the CKNN/US group. Although CKPB/US showed a moderate increase in CD206 protein expression versus CKNN/US, the immunofluorescence-based M2 proportion (CD206^+^/F4/80^+^ ratio) and the M1 marker CD86 showed no significant differences between the two treatment groups, supporting the conclusion that piezoelectric stimulation is the dominant driver of macrophage reprogramming.

### 2.7 KPB/US exhibited multifaceted protective effects in a model of radiation-induced injury *in vitro*

**Fig. 7:**
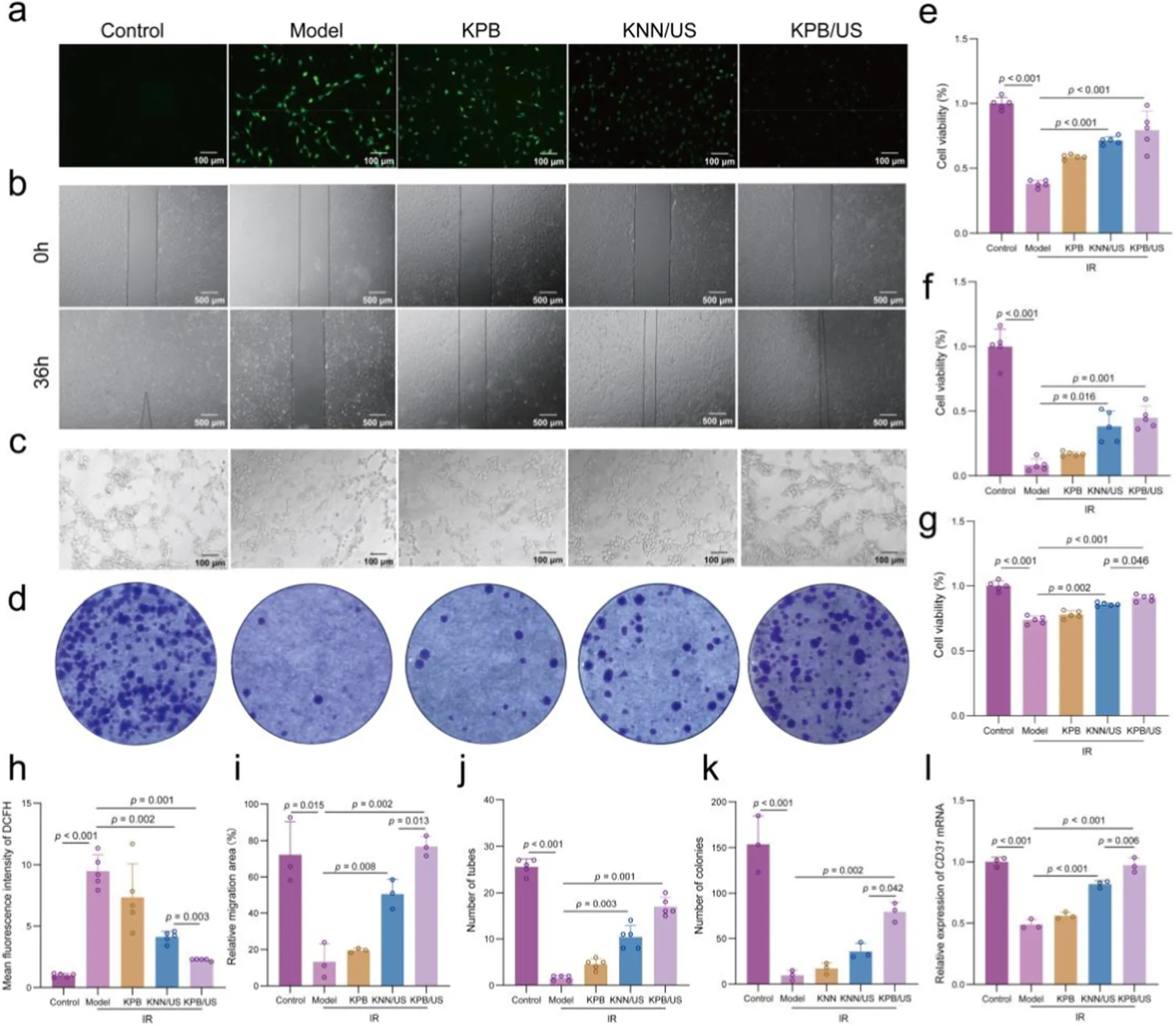
**KPB/US exhibited multifaceted protective effects in a model of radiation-induced injury *in vitro*.** (a) DCFH-DA fluorescence staining of HUVECs, indicating reduced ROS levels in the KPB/US group. Scale bar = 100 μm. (b) Scratch wound healing assay of HUVECs at 0 and 36 h post-treatment, indicating improved migration in the KPB/US group. Scale bar = 500 μm. (c) Representative images of tube formation assay of HUVECs indicating improved tube formation in the KPB/US group. Scale bar = 100 μm. (d) Colony formation assay of HUVECs indicating enhanced colony formation in the KPB/US group. (e) Quantification of WS1 cell viability after radiation injury (IR) indicating bioactive repair in the KPB/US group. (f) Quantification of HUVECs cell viability after radiation injury (IR) indicating bioactive repair in the KPB/US group. (g) Quantification of PC12 cell viability after radiation injury (IR) indicating bioactive repair in the KPB/US group. (h) Quantification of ROS relative fluorescence intensity (n = 5). (i) Quantification of migration area (n = 3). Data are presented as mean ± SD. (j) Quantification of colony numbers (n = 3). Data are presented as mean ± SD. (k) Quantification of tube formation (n = 3). Data are presented as mean ± SD. (l) Relative mRNA expression of CD31 (n = 3).

To further validate the therapeutic mechanisms observed *in vivo*, we conducted comprehensive *in vitro* analyses. Our results demonstrated that KPB/US significantly enhanced cell viability compared to the Model group in WS1 fibroblasts (0.904 ± 0.048 vs. 0.579 ± 0.055; *p* < 0.001; Fig. 7e), HUVECs (0.908 ± 0.025 vs. 0.739 ± 0.030; *p* < 0.001; Fig. 7f), and PC12 cells (0.450 ± 0.091 vs. 0.083 ± 0.048; *p* < 0.001; Fig. 7g). While KPB/US and KNN/US showed comparable efficacy in WS1 cells, KPB/US exhibited superior efficacy to KNN/US in both HUVECs (0.908 ± 0.025 vs. 0.860 ± 0.013; *p* < 0.05) and PC12 cells (0.450 ± 0.091 vs. 0.382 ± 0.120; *p* = 0.016). KPB/US also potently attenuated oxidative stress, as evidenced by a significant reduction in ROS levels in HUVECs compared to the Model group (*p* < 0.001; Fig. 7a–h). Moreover, KPB/US treatment effectively reduced the lipid peroxidation product MDA in WS1 cells (*p* < 0.001 vs. Model; Supplementary Fig. S5c), and outperformed KNN/US in restoring the activities of antioxidant enzymes (CAT: 0.895 ± 0.114 vs. 0.518 ± 0.037; *p* = 0.005; SOD: 0.762 ± 0.134 vs. 0.636 ± 0.078; *p* < 0.001; Supplementary Fig. S5a and b). Furthermore, KPB/US treatment significantly suppressed the radiation-induced upregulation of key pro-inflammatory cytokines in WS1 cells compared to the Model group; including IL-1β (1.097 ± 0.064 vs. 3.543 ± 0.377; *p* < 0.001; Supplementary Fig. S5d), IL-6 (1.286 ± 0.120 vs. 4.177 ± 0.625; *p* = 0.041; Supplementary Fig. S5e), and TNF-α (1.371 ± 0.131 vs. 5.117 ± 0.662; *p* < 0.001; Supplementary Fig. S5f), with a more pronounced inhibitory effect on IL-6 than KNN/US (1.286 ± 0.120 vs. 1.713 ± 0.090; *p* = 0.041; Supplementary Fig. S5g). In pro-angiogenic assays, KPB/US markedly improved HUVEC functions: it enhanced migration compared to both the Model group (76.79% ± 5.523% vs. 13.27% ± 9.792%; *p* = 0.001), and the KNN/US group (76.79% ± 5.523% vs. 50.63% ± 8.101%; *p* = 0.013; Fig. 7b–i), significantly promoted tube formation compared to the Model group (17.00 ± 2.000 vs. 1.600 ± 0.548; *p* = 0.001) with a stronger trend than KNN/US (10.40 ± 2.510; Fig. 7c–j), and increased colony formation (0.793 ± 0.106 vs. KNN/US: 0.360 ± 0.087; *p* = 0.042; Fig. 7d–k). At the molecular level, qPCR analysis confirmed that KPB/US significantly upregulated CD31 mRNA expression in HUVECs compared to both the Model group (0.972 ± 0.060 vs. 0.488 ± 0.040; *p* < 0.001) and the KNN/US group (0.972 ± 0.060 vs. 0.819 ± 0.028; *p* = 0.006; Fig. 7l). These *in vitro* findings corroborate the *in vivo* results; mechanistically demonstrating that KPB/US alleviates radiation-induced injury by promoting cell survival, reducing oxidative stress, suppressing inflammation, and enhancing angiogenic functions, with overall efficacy superior to KNN/US.

## 3 Discussion

The therapeutic breakthrough of the CKPB hydrogel for RUs repair lies in its paradigm-shifting integration of US-activated dual-functional therapies and targeted regulation of the neurovascular-immune homeostasis, addressing the core pathological complexity of RUs that has long confounded conventional therapies. Conventional electrical stimulation relies on external electrodes that are invasive, and incapable of addressing the multi-barrier pathology of RUs. NO therapy, despite its pleiotropic bioactivity, has been clinically hindered by uncontrolled release kinetics and consequent tissue toxicity [35]. The fundamental engineering challenge, therefore, is not merely combining these two modalities, but integrating them into a single platform that is noninvasive, spatiotemporally controllable, and capable of conforming to the complex topography of radiation-damaged dermal and subcutaneous tissue.

Functional materials represent a promising strategy for realizing such a platform [36,37] in the context of radiation-damaged tissue repair. Representative strategies include *in situ* electric stimulation [38], sustained NO release [39], and their combined application for nerve repair [40]. Herein, we fabricated CKPB hydrogel that an injectable, *in situ*-gelling collagen matrix from shad fish scales, with polydopamine anchoring NO donor BNN6 and KNN piezoelectric nanoparticles. It enabled ultrasound-triggered concurrent electric field, on-demand NO release, deep penetrability, and spatiotemporal control-transforming the passive filler into a wireless controlled therapeutic implant that operates beneath intact skin.

RUs are characterized by persistent inflammation, compromised vascular perfusion, and neuronal dysfunction. Complex crosstalk among immune cells, blood vessels and neurons maintains neurovascular-immune homeostasis, which modulates the wound healing network to drive tissue regeneration [15,41]. Specifically, macrophages act as central regulators and shape the inflammatory microenvironment via polarization toward pro-inflammatory M1 or pro-healing M2 phenotypes [42]. Blood vessels deliver nutrients and oxygen to support tissue repair. Neurons, in turn, modulate immune responses [9] and vascular remodeling through neurotransmitters [15]. Piezoelectric stimulation emerged as the dominant driver of nerve regeneration and macrophage reprogramming [43]. Thus, therapeutic strategies targeting the neurovascular-immune homeostasis are expected to achieve comprehensive RUs repair.

Our results revealed that the CKPB/US treatment rescued PGP9.5^+^ nerve fiber density and nNOS expression, suppressed CD86 and upregulated CD206 in RUs mice. Relative to CKNN/US, CKPB/US yield limited benefits on neuroimmune parameters and macrophage polarization, with only modestly increased CD206. By contrast, CKPB/US promoted therapeutically relevant angiogenesis via significant upregulation of CD31^+^ vessel density and eNOS expression. Meanwhile, it augmented the antioxidant defense, exhibiting superior ROS scavenging, decreased MDA level, and restored SOD and CAT activities. Comprehensively, the CKPB platform significantly restores radiation-damaged neural, vascular and immune homeostasis.

Critically, the hydrogel possessed favorable biocompatibility (hemolysis ratio < 5%; > 90% viability in fibroblasts, HUVECs, and neural precursors, Supplementary Fig. S6a–d) and injectability suitable for irregular defects. The platform confered potent antibacterial activity, lowering *S. aureus* CFUs by ∼95%. Since bacterial colonization of radiation-compromised tissue aggravates inflammation and necrosis, this intrinsic antimicrobial function distinguishes CKPB from bioinert hydrogel implants [44]. For patients with deep, irregular RUs or refractory infections ineligible for surgery, CKPB offers a viable alternative that bridges the gap between preclinical innovation and clinical requirements. Nevertheless, the long-term degradation behavior of KNN nanoparticles remains to be further explored. In addition, future studies involving large-animal models are warranted to advance the translational potential of this platform toward clinical applications.

## 4 Conclusion

In summary, we have developed an injectable, ultrasound-activated collagen hydrogel that integrates piezoelectric KNN nanoparticles loaded with a US-sensitive NO donor, enabling synchronous, wireless generation of localized electrical stimulation and spatiotemporally controlled NO release for radiation ulcer therapy. The hydrogel's injectability permits minimally invasive delivery, its US-responsiveness achieves noninvasive, on-demand activation directly within the damaged microenvironment, and its intrinsic antibacterial activity addresses the infection burden that frequently exacerbates radiation-induced tissue destruction. The CKPB platform facilitates nerve regeneration and M2 macrophage polarization, while enhancing angiogenesis and antioxidant defense. These effects yield favorable therapeutic outcomes, manifested by restored near-physiological epidermal architecture, improved functional blood perfusion, and enhanced overall tissue integrity. This work establishes a clinically translatable, noninvasive platform to treat pathological progression in RUs. It further offers a rational design paradigm for integrating diverse therapeutic cues within a single stimuli-responsive implantable system.

## 5 Materials and methods

### 5.1 Materials

#### 5.1.1 Reagents and materials

Shad collagen, primarily type I, was extracted from shad fish scales sourced from Suzhou Fish seeds Biotechnology (Suzhou, China). Its composition was confirmed by Coomassie Brilliant Blue staining, revealing α1, α2, β, and γ chains (Supplementary Fig. S6e). The facility operates under sustainable recirculating aquaculture systems (RAS) with traceable safety management protocols.

NN’-Di-sec-butyl-NN’-dinitroso-1,4-phenylene diamine (BNN6, HWRK CHEM, China; Cat. No. HWG60573; CAS 106476-75-9; purity 95%; molecular weight 278.35; appearance: white-to-grey powder) and dopamine hydrochloride (pDA, Aladdin, China; Cat. No. D103111-25g; Lot I12328217; CAS 62-31-7; purity 98%; molecular weight 189.64) were used as received.

The following reagents were used: fetal bovine serum (A5256701, Gibco, USA); Dulbecco's Modified Eagle Medium high-glucose (PM150210, Pricella, China); phosphate-buffered saline (bl302a, Biosharp, China); penicillin-streptomycin (C0222, Beyotime, China); 0.25% pancreatic enzyme cell digestate (C0201, Beyotime, China); Crystal Violet Staining Solution (C0121, Beyotime, China); dimethyl sulfoxide (D8371, Solarbio, China); dihydroethidium (HY-D0079, MCE, USA); Cell Counting Kit-8 (C0038, Beyotime, China); Total Superoxide Dismutase Assay Kit with WST-8 (S0101S, Beyotime, China); Lipid Peroxidation (MDA) Assay Kit (S0131S, Beyotime, China); Enhanced BCA Protein Assay Kit (P0010S, Beyotime, China); Catalase Assay Kit (S0051, Beyotime, China); 4% paraformaldehyde (BL539A, Biosharp, China); Total Nitric Oxide Assay Kit (S0024, Beyotime, China); hematoxylin-eosin staining kit (G1120, Solarbio, China); Masson staining kit (G1340, Solarbio, China); Trizol reagent (R711-01, Vazyme, China); HiScript III RT Super Mix (R323-01, Vazyme, China); and Cham SYBR qPCR Master Mix (Q711-02, Vazyme, China).

Antibodies included:Anti-CD31 antibody (1:100, ab227656, Abcam); Anti-GAPDH antibody (1:1000, ab9485, Abcam); Anti-eNOS antibody for IF (1:50, 27120-1-AP, Proteintech) and for WB (1:1000, 27120-1-AP, Proteintech); Anti-iNOS antibody for IF (1:100, 22226-1-AP, Proteintech) and for WB (1:1000, 22226-1-AP, Proteintech); Anti-nNOS antibody for IF (1:50, 29231-1-AP, Proteintech) and for WB (1:1000, 29231-1-AP, Proteintech); Anti-PGP9.5 antibody for IF (1:50, 14730-1-AP, Proteintech), for ICC (1:300, 14730-1-AP, Proteintech), and for WB (1:5000, 14730-1-AP, Proteintech); Anti-CD206 antibody for IF (1:100, TU313804, Abmart) and for WB (1:2000, TU313804, Abmart); Anti-F4/80 antibody (1:50, P47789, Abmart); Anti-Tuj-1 antibody (1:50, 66375-1-Ig, Proteintech).

#### 5.1.2 Cells and animals

Human skin fibroblast cells (WS1) were obtained from ATCC (CRL-1502). Umbilical vein endothelial cells (HUVECs) were provided by Professor Li Jianxiang's group (Soochow University), and rat pheochromocytoma cells (PC12) were provided by Dr. Zhu Zhanchi (Suzhou Institute of Nanotech and Nano-Bionics, Chinese Academy of Sciences). Male Balb/c mice (SPF, 6–8 weeks old) were obtained from the Laboratory Animal Center of Soochow University. All animal experiments were approved by the Animal Ethics Committee of Soochow University (SUDA20230619A04) and conducted in accordance with relevant guidelines.

#### 5.1.3 Instruments and equipment

The following instruments were used: inverted microscope (CKX53, Olympus, Japan); confocal imaging system (A1R, Nikon, Japan); multi-mode plate reader (FL600, Biotek, USA); FTIR spectrometer (NEXUS 670, Thermo, USA); fluorescence imaging system (ChemiDoc MP, Bio-Rad, USA); X-ray irradiator for biological studies (23 EX, Varian, USA); laser Doppler flowmetry device (MoorLDI2, Moor Instruments, UK); commercial transducer (DYW-1M, 1 MHz, China); function generator (RIGOL-DG812, China); ultrasonic cleaning device (JY92-IIN, Xin Zhi Biology, China); qPCR machine (QuantStudio 5, Thermo, USA); optical microscope (Eclipse E100, Nikon, Japan); image analysis software (Image-Pro Plus 6, Media Cybernetics, USA); electrophoresis equipment (Mini-PROTEAN Tetra, Bio-Rad, USA); wet transfer system (Trans-Blot SD, Bio-Rad, USA); rotational rheometer (Kinexus, Malvern, UK); gel permeation chromatography system (Agilent Technologies, USA); and XPS instrument (Thermo Fisher Scientific, USA).

### 5.2 Experimental section

#### 5.2.1 Synthesis of col hydrogel

Shad fish scales, pre-cleaned with 0.5% SDS, were treated with 0.5 M NaOH at 4 °C for 24 h, followed by immersion in 0.5 M EDTA for 48 h to remove calcium. The decalcified scales were dried, crushed into powder, and extracted with 0.5 M acetic acid to solubilize collagen. The collagen solution was then salted out using 0.9 M NaCl, centrifuged at 10,000 × g for 30 min at 4 °C, and the precipitate was collected. Finally, the collagen was purified by dialysis (14 kDa cutoff), freeze-dried, and stored at −20 °C.

#### 5.2.2 Synthesis of KNN, KP, and KPB

The hydrothermal synthesis of potassium sodium niobate (KNN) powder is conducted by first finely grinding niobium pentoxide (Nb_2_O_5_) to improve its reactivity. This powder is then placed into a PTFE-lined autoclave along with a cooled, highly concentrated aqueous solution of potassium and sodium hydroxides, with the total concentration typically between 6 and 7 M and a KOH to NaOH ratio designed. The sealed autoclave is heated in an oven at a controlled temperature, such as 200 °C, for a sustained period, commonly 24 h, to facilitate crystallization. After the reaction is complete and the vessel has cooled naturally, the resulting product is collected, then repeatedly washed with deionized water via centrifugation until the supernatant reaches a neutral pH to remove residual alkali, followed by a final ethanol wash. The purified powder is subsequently dried in a vacuum oven at 60-80 °C to yield the final crystalline KNN product.

The KP nanocomposite particles were synthesized by uniformly dispersing the as-prepared KNN powder in 0.1 M Tris-HCl buffer, followed by adding an equal volume of pDA solution. The mixture was ultrasonicated for 30 min, resulting in a characteristic greyish-black suspension. After stirring at 4 °C for 12 h to complete the polymerization coating, the product was collected by centrifugation (12,000 rpm, 10 min, 4 °C). The pellet was washed three times with PBS buffer via centrifugation under the same conditions to remove unbound residues. Finally, the purified precipitate was freeze-dried to obtain the KP nanoparticles.

BNN6 was dissolved in DMSO and blended with pre-prepared KP. After 24 h dark stirring and 12 h static incubation, the mixture was centrifuged at 12,000 rpm (4 °C, 10 min). The harvested precipitate was freeze-dried to yield KPB nanoparticles.

#### 5.2.3 Synthesis of CKNN, and KPB

Col was dissolved in ddH_2_O to achieve a concentration of 0.5% (w/v) and allowed to hydrate under continuous stirring at 4 °C for 24 h. The fully dissolved collagen solution was then uniformly mixed with the as-prepared KNN or KPB powders, respectively, to form the final composites denoted as CKNN and CKPB. KPB was used for *in vitro* cell experiments, while CKNN and CKPB were applied for *in vivo* animal studies.

#### 5.2.4 Characterization of KPB

To systematically characterize the physicochemical properties of the nanoparticles, their morphology was first examined using high-resolution field emission scanning electron microscopy. Prior to observation, the samples were mounted on a stage coated with conductive adhesive and sputter-coated with gold for 1 min in a vacuum coater. Subsequently, the crystalline phases were identified by X-ray diffraction with 2θ scanning from 10° to 80°. The surface elemental composition and chemical states were analyzed via X-ray photoelectron spectroscopy. Additionally, the thermal stability and weight loss profile of the sample were evaluated through thermogravimetric analysis.

#### 5.2.5 Measurement of piezo response of KPB under ultrasound

The ultrasound system included a commercial transducer, a function generator, and a power amplifier (custom-built, fabricated depending on the frequency of the commercial transducer) as we previously reported [25]. The piezoelectric output of the KPB nanoparticles in response to ultrasound was quantified. A suspension of the nanoparticles (5 mg/mL in PBS) was pipetted into the microwells of a silicone membrane. Controlled acoustic stimulation was then delivered by the ultrasound transducer positioned beneath the membrane, applying impulses with a frequence of 800 kHz, voltage of 10 Vpp, and duration of 10 ms at 100 cycles. For electrical measurement, bipolar electrodes were immersed into the suspension, and the generated voltage signals were captured and recorded via a high-resolution digital oscilloscope (UPO2202, UNIT) using shielded connecting cables.

#### 5.2.6 NO release of KPB under ultrasound

NO release from the nanoparticles under ultrasound was evaluated both *in vitro* and *in vivo*. For the *in vitro* assessment, nanoparticles (1 mg) dispersed in PBS were exposed to continuous ultrasound (800 kHz, 10 Vpp, 10 ms impulse, 3000 cycles) in an ultrasonic bath, and the amount of NO released into the supernatant was determined using a commercial Griess assay kit. For vivo experiments, the nanoparticles were co-injected with the fluorescent NO probe DAC-S into the subcutaneous layer of mouse skin. Following a 10-min period of ultrasound stimulation (800 kHz, 10 Vpp, 10 ms pulse interval, 3000 cycles), real-time NO production was visualized using an *in vivo* imaging system (IVIS 200, PerkinElmer). Subsequently, the treated skin tissues were harvested, homogenized, and the NO content within the lysates was also quantified via the Griess assay method.

#### 5.2.7 Cell culture

Lyophilized KNN and KPB powders were sterilized under UV light for 1 h. KNN was suspended in DMEM complete medium to achieve a concentration of 100 μg/mL, and KPB was similarly suspended at 100 μg/mL. The experimental design included the following groups: Control, Model, KPB (without ultrasound), KNN/US (KNN with ultrasound), and KPB/US (KPB with ultrasound). All groups except the Control group were subjected to 20 Gy of X-ray irradiation.

#### 5.2.8 Cell viability assay (CCK-8)

WS1, HUVECs, and PC12 cells in the logarithmic growth phase were trypsinized, resuspended, and seeded into 96-well plates at a density of 5 × 10^3^ to 1 × 10^4^ cells per well. Upon reaching 70-80% confluence, the cells were irradiated with 20 Gy of X-rays. Following irradiation, each treatment group received the corresponding interventions. For the ultrasound-treated groups, cells were exposed to ultrasound for 15 min daily for two consecutive days. After a total incubation period of 48 h, the cells were washed twice with PBS and then incubated with 100 μL of CCK-8 solution (diluted 1:10 in medium) for 2 h. Finally, the optical density (OD) at 450 nm was measured using a microplate reader.

#### 5.2.9 Colony formation assay

HUVECs at approximately 70% confluence were irradiated with X-rays and then cultured in DMEM complete medium. The medium was supplemented with 100 μg/mL of KNN, KPB, or sterile PBS as a vehicle control. For the groups designated to receive ultrasound (KNN/US and KPB/US), cells were subjected to ultrasound treatment for 15 min every two days. Following a 14-day culture period, the cells were fixed with 4% paraformaldehyde for 15 min and stained with 0.1% crystal violet for 30 min. Colonies consisting of more than 50 cells were counted manually, and the number of clones exceeding 50 μm in diameter was quantified using ImageJ software.

#### 5.2.10 Tube formation assay

HUVECs in the logarithmic growth phase were trypsinized, counted, and seeded into 6-well plates. After reaching appropriate confluence, the cells were irradiated with 20 Gy of X-rays and subsequently subjected to the corresponding treatments. Specifically, cells in the ultrasound-treated groups (KNN/US and KPB/US) were exposed to ultrasound for 15 min per day for two consecutive days. Following treatment, the cells were harvested for the tube formation assay. Matrigel was thawed overnight at 4 °C, and 50 μL was carefully plated into each well of a 48-well plate. The plates were then incubated at 37 °C for 30 min to allow the Matrigel to polymerize. The pretreated HUVECs were resuspended in serum-free medium at a density of 1 × 10^6^ cells/mL. A 100 μL aliquot of the cell suspension was added onto the surface of the polymerized Matrigel in each well. The plates were incubated at 37 °C in a 5% CO_2_ humidified incubator for 4-8 h. Tube formation was observed under an inverted microscope, and the number of enclosed lumens (tube-like structures) in three random fields per well was analyzed using ImageJ software to quantitatively assess the angiogenic capacity of the cells.

#### 5.2.11 Staphylococcus aureus antimicrobial assay

Briefly, *S. aureus* cultures in the logarithmic growth phase were harvested, centrifuged, and resuspended in sterile PBS to standardize the bacterial suspension. The suspension was then divided into four treatment groups: Control (bacteria alone), KPB (bacteria with KPB composite), KNN/US (bacteria with KNN composite followed by ultrasound treatment), and KPB/US (bacteria with KPB composite followed by ultrasound treatment). For the ultrasound-treated groups (KNN/US and KPB/US), the mixtures were subjected to ultrasonic irradiation for 30 min. Subsequently, a 100 μL aliquot from each group was serially diluted, and a dilution corresponding to approximately 1 × 10^6^ CFU/mL was spread onto solid agar plates. All plates were incubated at 37 °C for 12 h. After incubation, the number of viable bacterial colonies on each plate was counted manually, and the data were expressed as colony-forming units (CFU) for statistical analysis to determine the antibacterial efficacy of the different treatments. The antibacterial efficiency (%) was calculated according to the formula: Antibacterial rate (%) = [(CFU count of control group − CFU count of experimental group)/CFU count of control group] × 100%.

#### 5.2.12 *In vivo* evaluation of radiation-induced skin injury repair

All animal procedures were approved by the Animal Ethics Committee of Soochow University and conducted in accordance with relevant guidelines. Eight-week-old female BALB/c mice were randomly divided into five groups (n = 5 per group): (1) Sham (no irradiation or injection); (2) Vehicle (irradiation + PBS injection); (3) KPB (irradiation + CKPB injection); (4) CKNN/US (irradiation + CKNN injection + ultrasound); (5) KPB/US (irradiation + KPB injection + ultrasound). As established in our previous work, the concentration of collagen (Col) was fixed at 0.5% (w/v), while KNN and KPB were used at 5 mg/mL. Mice in all groups except the Sham group received local irradiation of both hind limbs with a total absorbed dose of 45 Gy electron beam (500 cGy/min), with non-target areas shielded by a 3 cm thick lead plate. On the day following irradiation, 200 μL of the respective test mixture or PBS was injected subcutaneously into the hind limbs. Mice in the KNN/US and KPB/US groups received daily ultrasound treatment for 10 min at 24-h intervals. The severity of skin damage was assessed by two independent, blinded observers on days 5, 10, 15, and 20 post-irradiations using a standardized radiodermatitis scoring scale (RTOG scale). Evaluations were based on ulcer area, erythema, and ulcer healing progression, with photographic documentation at each time point. All mice were euthanized on day 21, and skin samples from the irradiated sites were collected for further analysis.

#### 5.2.13 Doppler ultrasound blood flow analysis

On day 21post-irradiation, mice were anesthetized via intraperitoneal injection of 0.5% sodium pentobarbital (50 mg/kg). Local blood perfusion in the hind limbs was then scanned and quantified using a laser Doppler imager. Blood flow measurement served as a functional indicator of angiogenesis and microvascular recovery.

#### 5.2.14 Histology, immunohistochemistry (IHC), and immunofluorescence (IF)

Collected skin tissues were fixed in 4% paraformaldehyde for 24 h, followed by dehydration, paraffin embedding, and sectioning at 4 μm thickness. Sections were stained with HE and Masson's trichrome according to standard protocols. For IHC and IF, sections were dewaxed, and antigen retrieval was performed by heating in sodium citrate buffer (95 °C, 20 min). After blocking with 3% H_2_O_2_ (10 min) and 5% bovine serum albumin (BSA, 30 min), sections were incubated overnight at 4 °C with primary antibodies (e.g. anti-CD31, anti-VEGF). For IHC, an HRP-conjugated secondary antibody was applied for 1 h, followed by DAB development and hematoxylin counterstaining. A semi-quantitative H-Score, calculated as the product of staining intensity and the percentage of positive cells, was determined. For all histological and IHC analyses, three random fields per sample were evaluated in a blinded manner using ImageJ software (NIH). Data are presented as the mean ± standard deviation from five biological replicates.

#### 5.2.15 ROS staining

*In vivo*: Skin tissues fixed in 4% PFA were dehydrated in 20% and 30% sucrose solutions, embedded in OCT compound, and sectioned at 10 μm. Sections were incubated with 1.5 μM dihydroethidium at 37 °C for 30-60 min, washed with PBS, and mounted with an anti-fade medium. ROS distribution and fluorescence intensity were examined and quantified using fluorescence microscopy.

*In vitro* (WS1 cells): Cells in the logarithmic growth phase were seeded in 6-well plates at 5 × 10^5^ cells/well and cultured until 70-80% confluence. After irradiation and designated treatments (e.g. a 10-min ultrasound for the KNN/US and KPB/US groups), cells were incubated for an additional 24 h. Subsequently, cells were washed with PBS and loaded with 10 μM DCFH-DA probe (diluted in serum-free medium) in the dark for 20-30 min at 37 °C. After washing three times with PBS to remove excess probe, intracellular ROS levels were assessed by measuring fluorescence intensity (Ex/Em: 488/525 nm) using fluorescence microscopy.

#### 5.2.16 Antioxidant capacity assessment

The activities of SOD and CAT, as well as the level of MDA, were measured using commercial assay kits. Tissue samples (about 30 mg) were homogenized in the provided buffer, while WS1 cells in 6-well plates were lysed with 150 μL of lysis buffer. After centrifugation at 10,000 × g for 10 min at 4 °C, the supernatant was collected. The total protein concentration was normalized to 2 μg/μL using a BCA protein assay kit. SOD activity was determined at 535 nm, CAT activity at 520 nm, and MDA concentration was calculated based on the absorbance at 532 nm, following the respective kit protocols.

#### 5.2.17 Western blot analysis

Approximately 20 mg of skin tissue was homogenized in 180 μL of RIPA lysis buffer containing 1% PMSF. The homogenate was centrifuged at 12,000 × g for 15 min at 4 °C. The protein concentration of the supernatant was adjusted to 4 μg/μL using a BCA assay. Proteins were separated by 10% SDS-PAGE and transferred onto a PVDF membrane. After blocking with 5% skim milk, the membrane was incubated overnight at 4 °C with the primary antibody, followed by a 1 h incubation with an HRP-conjugated secondary antibody. Protein bands were visualized using an ECL substrate, and their intensities were quantified with ImageJ. GAPDH served as the loading control for normalization. The original data was showed in Supplementary Fig. S7.

#### 5.2.18 Quantitative real-time PCR (qRT-PCR)

Total RNA was extracted from cells or fresh tissues using Trizol reagent. RNA purity (A260/A280 ratio of 1.8-2.0) and concentration were verified with a Nanodrop spectrophotometer. CDNA was synthesized from 1 μg of total RNA using a reverse transcription kit. qPCR was performed with gene-specific primers and a SYBR Green master mix on a real-time PCR system. The thermal cycling conditions were initial denaturation at 95 °C for 30 s, followed by 40 cycles of 95 °C for 5 s and 60 °C for 30 s. All reactions were performed in triplicate, including no-template controls. The relative expression of target genes was calculated using the 2^^(−ΔΔCt)^ method, normalized to Gapdh (for cells) or Actb (for tissues).

### 5.3 Statistical analysis

All experiments were performed with at least three independent replicates. Data are presented as mean ± standard deviation (SD). Statistical analysis was conducted using GraphPad Prism 10. Normality was assessed with the Shapiro-Wilk test. For comparisons between two groups, an unpaired two-tailed Student's t-test (normal distribution) or the Mann-Whitney *U* test (non-normal distribution) was used. For comparisons among multiple groups, one-way or two-way ANOVA followed by Tukey's post hoc test was applied for data with normal distribution and homogeneity of variance; otherwise, the Kruskal-Wallis test with Dunn's post hoc test was used. A p-value of less than 0.05 was considered statistically significant.

## CRediT authorship contribution statement

**Yueyue Nie:** Conceptualization, Data curation, Investigation, Writing – original draft. **Xiaotong Zhao:** Data curation, Investigation, Writing – original draft. **Jingyi Li:** Data curation, Investigation, Validation, Writing – review & editing. **Xinyu Huang:** Investigation. **Biao Song:** Investigation. **Manlin Xu:** Investigation. **Zesu Wang:** Investigation. **Peifeng Zhao:** Investigation. **Beini Sheng:** Investigation. **Yan Wang:** Methodology. **Yali Jiang:** Investigation. **Mingliang Tang:** Conceptualization, Funding acquisition, Writing – review & editing. **Hongmei Tang:** Funding acquisition, Validation, Writing – review & editing. **Jihua Nie:** Project administration, Supervision, Writing – review & editing. **Miao Xiao:** Conceptualization, Funding acquisition, Methodology, Supervision, Validation, Writing – original draft.

## Declaration of competing interests

The authors declare that they have no known competing financial interests or personal relationships that could have appeared to influence the work reported in this paper.

## Data Availability

Data will be made available on request.
