# Supplementary material for: Spatiotemporal ultrasound-driven piezoelectric electro-NO therapy restores neuro-vascular-immune homeostasis for radiation ulcer repair
Source: Mater Today Bio. 2026 Aug 18;40:103581. doi: 10.1016/j.mtbio.2026.103581 (PMC13521218; doi:10.1016/j.mtbio.2026.103581)
Supplement: Supplementary file 1 [file mmc1.docx]

**Supplementary materials**

**Spatiotemporal Ultrasound-Driven Piezoelectric Electro-NO Therapy Restores Neuro-Vascular-Immune Homeostasis for Radiation Ulcer Repair**

Yueyue Nie ^1, 2, #^, Xiaotong Zhao ^1, #^, Jingyi Li ^1, #^, Xinyu Huang ^2^, Biao Song ^1^, Manlin Xu ^2^, Zesu Wang ^2^, Peifeng Zhao ^4^, Beini Sheng ^1^, Yan Wang ^2^, Yali Jiang ^2^, Mingliang Tang ^1, *^, Hongmei Tang ^3, *^, Jihua Nie ^2, *^, Miao Xiao ^1, *^

^1^Department of Cardiovascular Surgery of the First Affiliated Hospital & Institute for Cardiovascular Science, Suzhou Medical College, Soochow University, Suzhou, 215000, China.

^2^Department of Toxicology, School of Public Health, Medical College of Soochow University, Suzhou, 215000, China.

^3^Department of Plastic and Aesthetic surgery, the Second Affiliated Hospital of Soochow University, Suzhou Medical College, Soochow University, Suzhou, 215000, China.

^4^Department of Radiotherapy and Oncology, The Second Affiliated Hospital of Soochow University, Suzhou, 215000, China.

#These authors contributed equally to this work.

Emails: [mxiao@suda.edu.cn](mailto:mxiao@suda.edu.cn), [niejihua@suda.edu.cn](mailto:niejihua@suda.edu.cn), [tanghongmei1979@suda.edu.cn](mailto:tanghongmei1979@suda.edu.cn), [mltang@suda.edu.cn](mailto:mltang@suda.edu.cn)

**Figure S1. Subcutaneous in situ gel formation of CKNN and CKPB hydrogels.**

**Figure S2. Schematic of the ultrasound stimulation system for in vivo experiments.**

**Figure S3. Evaluation in ultrasound-only treatment in RUs mice.** (a) Representative images of murine hindlimb in vehicle and vehicle/US groups. (b) Clinical pathology scores over the 20-day period (n = 6). (c) Representative color Doppler imaging acquired at day 20. (d) Quantitative analysis of mean blood flow flux (n = 5). Data are presented as mean ± SD. ns, not statistically significant.

**Figure S4. *In vivo* long‑term NO release performance of CKPB hydrogel under ultrasound stimulation.** (a) Representative images of mice implanted with CKPB hydrogel at days 0, 5, 7, 14 and 21. (b) Quantitative analysis of *in vivo* NO concentration detected by NO assay kits. (c) Photograph showing the hydrogel location at day 21. (d) The measurement of NO signals triggered by ultrasound at days 7, 14 and 21. Data are presented as mean ± SD. ****p* < 0.001.

**Figure S5. qRT-PCR analysis of antioxidant-related indices and inflammatory factor expression in WS1 cells after irradiation.** (a) Relative SOD activity (%) (n = 5). (b) Relative CAT activity (%) (n = 5). (c) The level of MDA (nmol/mg prot) (n = 5). Date are presented as mean ± SD. These experiments were performed to verify the ability of KPB/US to reduce oxidative stress levels. (d) Relative mRNA expression of IL-1β (n = 3). (e) Relative mRNA expression of TNF-α (n = 3). (f) Relative mRNA expression of NF-κB (n = 3). (g) Relative mRNA expression of IL-6 (n = 3). These experiments were conducted to validate the anti-inflammatory effect of KPB/US.

**Figure S6. Biocompatibility evaluation, hydrodynamic particle size analysis, and protein electrophoresis results.** (a) Live/dead staining images of WS1, HUVEC, and PC12 cells. Scale bar = 100 μm. (b) Hemolysis assay showing the hemolysis ratio of the control group and KPB group; H2O was used as a positive control (n = 5). (c) CCK-8 assay results for cell viability of WS1, HUVEC, and PC12 cells (n = 5). (d) HE stains of heart, liver, spleen, lung, and kidney tissues from mice in the control group and CKPB/US group, confirming the biocompatibility of the material; Scale bar = 1mm. (e) Sodium Dodecyl Sulfate-Polyacrylamide Gel Electrophoresis (SDS-PAGE) analysis followed by Coomassie Brilliant Blue staining. The protein molecular weight markers (α1, α2, β, γ) are labeled with corresponding molecular weights (250 kDa, 150 kDa, 100 kDa, 70 kDa, 50 kDa).

**Figure S7. Original Western blot (WB) images of different target proteins.** (a) WB results for eNOS and GAPDH used as a loading control. (b) WB results for nNOS, PGP9.5 and GAPDH. (c) WB results for CD206, iNOS and GAPDH.
